# Treating hematological malignancies with drugs inhibiting ribosome biogenesis: when and why

**DOI:** 10.1186/s13045-018-0609-1

**Published:** 2018-05-31

**Authors:** Enrico Derenzini, Alessandra Rossi, Davide Treré

**Affiliations:** 1 European Institute of Oncology, Via Ripamonti 435, 20141 Milan, Italy; 2 DIMES, Università di Bologna, Via Massarenti 9, Bologna, Italy

**Keywords:** Ribosome biogenesis inhibitors, Chemotherapy, Lymphoma, Leukemia, Ribosomal proteins, MDM2, p53, pRb

## Abstract

It is well known that chemotherapy can cure only some cancers in advanced stage, mostly those with an intact p53 pathway. Hematological cancers such as lymphoma and certain forms of leukemia are paradigmatic examples of such scenario. Recent evidence indicates that the efficacy of many of the alkylating and intercalating agents, antimetabolites, topoisomerase, and kinase inhibitors used in cancer therapy is largely due to p53 stabilization and activation consequent to the inhibition of ribosome biogenesis. In this context, innovative drugs specifically hindering ribosome biogenesis showed preclinical activity and are currently in early clinical development in hematological malignancies. The mechanism of p53 stabilization after ribosome biogenesis inhibition is a multistep process, depending on specific factors that can be altered in tumor cells, which can affect the antitumor efficacy of ribosome biogenesis inhibitors (RiBi). In the present review, the basic mechanisms underlying the anticancer activity of RiBi are discussed based on the evidence deriving from available preclinical and clinical studies, with the purpose of defining when and why the treatment with drugs inhibiting ribosomal biogenesis could be highly effective in hematological malignancies.

## Background

The ribosome biogenesis is defined as the process of building new ribosomes, the intracellular organelles where protein synthesis takes place.

In recent years, several studies on the relationship between cell growth and proliferation produced important data regarding the mechanisms linking ribosome biogenesis, which is at the basis of cell growth, to the progression through the cell cycle phases of the proliferating cell. There is now evidence that a perturbed ribosome biogenesis activates a pathway leading to the stabilization and activation of the tumor suppressor protein p53, which in turn induces cell cycle arrest and/or apoptotic cell death [1–4].

Current evidence indicates that inhibition of ribosome biogenesis represents a major mechanism by which many of the currently used chemotherapeutic drugs (alkylating and intercalating agents, antimetabolites, topoisomerase inhibitors) exert their cytotoxic activity on cancer cells [5, 6]. Importantly, a series of new drugs selectively hindering the transcription of ribosomal (r) RNA, thus inhibiting ribosome biogenesis without having genotoxic effects, have been proposed as a new therapeutic approach, based on p53 activation [7–12]*.*

However, it is known since long time that chemotherapy can cure only some cancers once they reach advanced stages. In fact, despite initial responses, the majority of metastatic solid tumors ultimately progress under chemotherapy treatment. Hematological malignancies (such as lymphomas and acute leukemias) represent paradigmatic examples of the few cancers that can be cured by chemotherapeutic agents and will be the main topic of the present review [13]. The basic biological characteristic underlying the intrinsic curability of such cancers is that, in a significant fraction of cases, they retain a functional p53-mediated response to nucleolar stress arising from ribosomal biogenesis inhibition; on the other hand, as a matter of fact, the presence of genomic alterations of the *TP53* gene is an established negative prognostic predictor in lymphoma, acute and chronic leukemias treated with chemotherapy regimens [14–17].

Since p53 stabilization and activation is a multistep and tightly regulated process, in principle, the prerequisite for the antitumor efficacy of drugs inhibiting ribosome biogenesis should be the presence in the tumor cells, other than a normally functioning p53, also of those factors necessary for the activation of p53 and the induction of a p53-mediated cell cycle arrest and/or the apoptosis. These factors, which control cell cycle progression in normally proliferating cells [18], are qualitatively and quantitatively altered in the large number of cancers [19, 20], thus influencing the sensitivity to ribosome biogenesis (RiBi) inhibitors.

Therefore, it seems timely to critically review the characteristics of cancer cells which affect their sensitivity to RiBi inhibitors, with the purpose of highlighting those parameters which render the treatment with these drugs appropriate or not in hematological malignancies. For the convenience of the reader, the normal process of ribosome biogenesis will be first briefly described.

## Ribosome biogenesis

Ribosomes are ribonucleoprotein particles which are located in the cytoplasm where, either free or membrane-bound, are engaged in protein synthesis. Four types of ribosomal RNA (rRNA) molecules and about 80 different ribosomal proteins constitute the ribosome. Ribosome formation occurs mainly in the nucleolus, being later completed in the nucleoplasm and in the cytoplasm (see for reviews: [21–24]). In the nucleolus, ribosomal genes are transcribed by RNA polymerase I (Pol I) to generate the 47S rRNA precursor, which undergoes to site-specific methylation and pseudo uridylation, and processing to give rise to the mature 18S, 5.8S, and 28S rRNA. The fourth types of rRNA, the 5S rRNA, is synthesized in the nucleoplasm by RNA polymerase III (Pol III) and then imported in the nucleolus together with the ribosomal proteins (RPs), whose mRNA is transcribed by RNA polymerase II (Pol II). The assembling of rRNA molecules with the RPs constitutes the two subunits of the mature ribosome, the large 60S and the small 40S subunit. The large 60S subunit is constituted by one each of the 28S, 5.8S, and 5S RNA molecules, together with 47 ribosomal proteins (RPLs); the small 40S subunit contains only one 18S RNA molecule and 33 ribosomal proteins (RPSs) [25, 26]. Both subunits migrate from the nucleolus to the cytoplasm where they form the 80S ribosome particle. In the process of ribosome biogenesis, more than 150 non-ribosomal proteins and around 70 small nucleolar RNAs are involved [27–32].

For the transcription of the of 47S pre-rRNA, the assembly of a specific multiprotein complex at the rDNA promoter containing Pol I is required. In this complex, three basal factors, termed transcription initiation factor I (TIF-I) A, selectivity factor 1 (SL1), and upstream binding factor (UBF), are present [33]. For the transcription of the 5S rRNA by Pol III, the transcription factors TFIIIC and TFIIIB are necessary [34–36]. In proliferating cells, the rate of ribosome biogenesis is enhanced in order to assure an adequate ribosome complement for the daughter cells and inhibition of ribosome biogenesis arrests cell cycle progression [37]. Furthermore, the rate of ribosome biogenesis influences the length of the cell cycle: higher the level of ribosome biogenesis, more rapid the cell cycle progression [38]. Ribosome biogenesis rate in cancer shows high variability, depending on a multiplicity of factors including the activation of specific intracellular signaling pathways and deregulated activity of oncogenes and tumor suppressors. On the other hand, quantitative and qualitative changes in ribosome biogenesis have been shown to facilitate neoplastic transformation. For a detailed description of the relationship between ribosome biogenesis and cancer, the reader should refer to [39–44]. In hematological malignancies, such as aggressive lymphoproliferative neoplasms, it is worth mentioning the oncogenic cooperation between the *MYC* oncogene and the phosphatidyl-inositol-3-kinase (PI3K) signaling pathway [45], which converge in stimulating rRNA synthesis and ribosome biogenesis [46].

## Inhibition of ribosome biogenesis activates the RPs/MDM2/p53 pathway

**Fig. 1:**
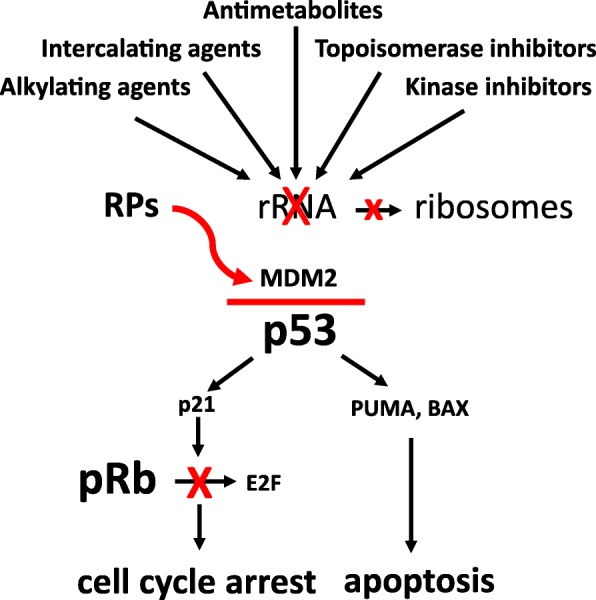
Schematic representation of the pathway activated by drug-induced perturbation of rRNA synthesis. Ribosomal proteins (RPs), no longer used for ribosome building, bind to MDM2, thus inhibiting its ubiquitin ligase activity toward p53 and the proteasome digestion of the tumor suppressor. As a consequence, p53 accumulates and induces transcription of p21, PUMA, and BAX. P21 is responsible for the cell cycle arrest by hindering pRb phosphorylation: in fact, hypo-phosphorylated pRb binds to and inhibits the activity of the transcription factor E2F1, whose target gene products are necessary for cell cycle progression. The induction of the pro-apoptotic factors PUMA and BAX activates the process of apoptotic cell death

Available data indicate that the levels of p53 expression and activity are mainly regulated by interactions with the tumor suppressor MDM2 (murine double minute 2, and HDM2 in humans). MDM2 is an E3 ubiquitin ligase which negatively controls p53 activity in two ways: by binding to the protein and inhibiting its transactivation activity, and by facilitating its proteasome degradation [47–49]. In normal proliferating cells, the level of p53 is maintained low because of the binding with MDM2 with consequent p53 ubiquitination and proteasome digestion [50]. When a perturbation in the ribosome biogenesis occurs (ribosome stress), it results in the binding of several ribosomal proteins, no longer used for ribosome building, to MDM2. This binding relieves the inhibitory activity of MDM2 toward p53 (see reviews [2–4, 51, 52]) (Fig. 1). Although there is evidence that RPL5, RPL11, and RPL23 play a major role in neutralization of MDM2 activity and in the induction of p53 stabilization [50, 53–58], the list of ribosomal proteins (of both large and small ribosomal subunit) able to inhibit MDM2 activity and to stabilize p53 upon “ribosomal stress” is rapidly expanding [52]. For a valid binding to MDM2 and its inactivation, the RPL11 and RPL5 must form a complex with the 5S rRNA and all the components of this complex are necessary for its inhibitory function [59, 60]. p53 stabilization always causes cell cycle arrest in proliferating cells and, depending on the quantitative level of stabilized p53, also apoptotic cell death [61–63]. p53 arrests cell cycle progression by inhibiting the phosphorylation of the tumor suppressor retinoblastoma protein, pRb. In its hypo-phosphorylated form, pRb binds to and inhibits the activity of E2F1, a transcription factor whose target genes are necessary for cell cycle progression. The inhibition of E2F1 activity by hypo-phosphorylated pRb reduces the expression of both cyclin E and A, necessary factors for cell cycle progression from G1 to S phase and from G2 to M phase respectively, with consequent cell accumulation in G1 and G2 phase [64]. The induction of apoptotic cell death by p53 is a consequence of induced expression of the pro-apoptotic members of the B cell lymphoma 2 (Bcl-2) gene family, PUMA, and BAX [63, 65–67] (Fig. 1). Finally, it should be noted that additional factors may interact with the RPs/MDM2/p53 axis, such as the ARF tumor suppressor and the activation of the PI3K pathway. In fact, ARF loss is a common genetic event in cancer and especially in aggressive lymphoid neoplasms, resulting in increased MDM2 activity and increased p53 degradation (reviewed in [68]). On the other hand, MDM2 is a downstream target of the PI3K-AKT axis, and AKT-induced MDM2 phosphorylation results in increased stability of MDM2 with consequent p53 degradation [69, 70]. As mentioned before, constitutive PI3K signaling is common in lymphoproliferative neoplasms, and PI3K inhibitors are in clinical development in lymphoid cancers. These notions could be relevant for designing therapeutic combination strategies aimed at increasing the p53-mediated response to the inhibition of ribosome biogenesis.

## Development of selective inhibitors of ribosome biogenesis

As briefly mentioned before, a strong contribution to p53 activation induced by chemotherapeutic agents is due to the inhibition of ribosomal biogenesis. As reported by Burger et al. [5], a series of drugs currently used for treating solid cancers and hematological malignancies inhibit ribosome biogenesis at the level of rRNA transcription and/or at the level of rRNA processing (Table 1). To this list, cyclophosphamide and mycophenolic acid should be added. Cyclophosphamide, a widely used anticancer drug, also inhibits rRNA transcription [71], after being converted to acrolein [72, 73], and the immunosuppressant mycophenolic acid has been demonstrated to inhibit the synthesis of rRNA [74].

**Table 1:** Drugs used to treat hematological and solid malignancies which are effective or highly effective in the inhibition of rRNA transcription or processing (modified from Burger et al., 2010) [5]

	inibition of rRNA synthesis	
	transcription	processing
Alkylating agents:		
Melphalan*	+	-
Cisplatin*	+	-
Oxaliplatin*	+	-
Cyclophosphamide ^1^ *	+	-
Intercalating agents:		
Doxorubicin *	+	-
Mitoxantrone *	+	-
Actinomycin D *	+	-
Mitomycin C	+	-
Antimetabolites:		
Methotrexate *	+	-
5-Fluorouracil	-	+
Topoisomerase inhibitors:		
Camptothecin	-	+
Etoposide*	-	+
Kinase inhibitors:		
Flavopiridol*	-	+
Roscovitine	-	+
Rapamycin	+	-
Proteasome inhibitors:		
Bortezomib*	-	+
Translation inhibitors:		
Homoharringtonine*	-	+
Mitosis inhibitors:		
Vinblastine*	-	+
rRNA polymerase I inhibitors:		
CX-5461 ^2^ *	+	-

* drugs currently used or in clinical development for the treatment of lymphomas and leukemia

^1^ Cyclophosphamide is metabolized to acrolein, which is responsible for the inhibition of rRNA transcription [60, 61]

^2^ CX-5461 is in phase I clinical trial in patients with haematological malignancies and in phase I/II trial in patients with breast cancer

In recent years, several efforts have been made to develop specific inhibitors of ribosomal biogenesis, in order to achieve a selective inhibition of rRNA synthesis without the genotoxic effects proper of chemotherapeutic drugs. In this light, it appears to be of particular relevance the CX-5461 molecule which selectively inhibits ribosome biogenesis, most likely by disrupting the SL-1/rDNA complex, promoting a cancer-specific activation of p53. Recent preclinical data indicate high activity of CX-5461 in MYC-driven lymphoma, providing the rationale for further clinical development of this compound [7, 75, 76]. CX-5361 is currently under phase I clinical trial for the treatment of patients with advanced hematologic malignancies, including acute myeloid leukemia.

Finally, there is experimental evidence that a small molecular compound, BMH-21, and a small-molecule peptide (22mer) also selectively inhibit rDNA transcription. BMH21 binds to GC-rich sequences and inhibits RNA Pol I activity [9]. It also induces the proteasome-dependent destruction of the large catalytic subunit in the Pol I complex, as do three other small molecular compounds, BMH-9, BMH-22, and BMH-23 [10]. The 22mer targets the interface between RNA polymerase I and Rrn3, thus selectively inhibiting the synthesis of rRNA [11].

## Factors determining cancer cell sensitivity to drugs inhibiting ribosome biogenesis

### The p53 status

Since a major effect of ribosome biogenesis inhibition is the activation of p53, the cytostatic and cytotoxic effects of chemotherapeutic agents inhibiting ribosome biogenesis should be obviously affected by the status of p53 [64, 77, 78]. Several lines of preclinical and clinical evidence support this notion. Indeed, actinomycin D, at a dose that exclusively hinders rDNA transcription, induced a cell cycle arrest with cell accumulation in G1 and, to a lesser extent, in G2 phase in p53 proficient cell lines [38, 64] whereas these changes in cell cycle distribution appeared to be reduced if cells were previously silenced for p53 expression [64]. The same occurs in cells with inactivated p53 in which the synthesis of rRNA was hindered by polymerase I silencing [79]. Also, p53 silencing significantly reduced the antiproliferative effects of 5-fluorouracil and methotrexate or doxorubicin, in human cancer cell lines harboring wild type (wt) p53 [78] and treatment of human leukemia and lymphoma cell lines with CX-5461, a selective inhibitor of Pol I transcription [7], was much more effective in cells with wt p53 in comparison with those with mutated p53 [75, 79].

On the other hand, it is worth noting that although p53 stabilization appears to be the main mechanism by which inhibitors of ribosomal biogenesis exert their cytostatic and cytotoxic action, there is evidence that these effects can be also caused in a p53-independent way. Depletion of the catalytic subunit of RNA polymerase I inhibited the synthesis of rRNA and hindered cell cycle progression in cells with inactivated p53, as a consequence of downregulation of the transcription factor E2F-1. Downregulation of E2F-1 was due to release of the ribosomal protein L11, which inactivated the E2F-1-stabilizing function of the E3 ubiquitin protein ligase MDM2 [79]. Furthermore, CX-5461 can induce p53-independent G2 checkpoint and apoptosis through activation of the ataxia telangiectasia mutated (ATM) and ataxia telangiectasia and Rad3-related (ATR) kinase pathway, in the absence of DNA damage [80, 81].

Regarding hematological malignancies, there is evidence that p53 status is an important factor determining the response to currently used chemotherapy regimens for lymphoma and leukemia treatment, which are based on drugs hindering ribosome biogenesis [5]. Anthracycline-based polychemotherapy represents the standard therapeutic approach for pediatric acute lymphoblastic leukemia (ALL) and multiple lymphoma subtypes of the adult [including Hodgkin lymphoma (HL), diffuse large B cell lymphoma (DLBCL), and anaplastic large T cell lymphoma (ALCL)]. More in detail, the ABVD (doxorubicin, bleomycin, vinblastine, and dacarbazine) and the CHOP (cyclophosphamide, doxorubicin, vincristine, and prednisone) regimens represent the treatments of choice in HL, DLBCL, and ALCL respectively. In general, the cure rates of antracycline-based regimens have been proved to be variable, being high for pediatric ALL and Hodgkin lymphoma [82, 83], intermediate for DLBCL [84–86] and ALCL [87, 88], and low for in indolent B cell lymphoma [89]. Similar considerations apply for myeloid disorders where anthracycline-based polychemotherapy has been shown to be effective certain forms of acute myeloid leukemia (reviewed in [90–92]), whereas chronic myeloid neoplasms are considered virtually incurable with standard polychemotherapy (reviewed in [93]). The intrinsic curability of the aforementioned hematologic cancers relies on precise biological characteristics of cancer cells, and the p53 status has been demonstrated to represent an important prognostic factor.

In line with this concept, the presence of *TP53* genomic alterations in DLBCL and chronic lymphoid leukemia is a well-established negative prognostic predictor [14, 16, 94, 95]. DLBCL harboring alterations of the p53 pathway are often nonresponsive to CHOP plus rituximab (R) chemoimmunotherapy and are characterized by shorter overall survival. In CLL, patients harboring 17p deletions or *TP53* mutations are refractory to standard chemotherapy and are currently treated with chemo-free treatments including inhibitors of B cell receptor signaling or bcl-2 inhibitors [96]. In acute myeloid leukemia, the presence of *TP53* mutations is a powerful negative prognostic predictor, being associated with refractoriness to current anthracycline-based induction therapies [92, 97]. Finally, the presence of *TP53* gene mutations predicts the outcome after induction and reinduction chemotherapy in acute lymphoid leukemia [98].

The prognostic value of genomic alterations of *TP53* has been recently evaluated across a wide variety of hematological malignancies confirming the role of the p53 axis in determining the efficacy of chemotherapy in this setting [15].

### The pRb status

These experimental and clinical data indicate that wild-type *TP53* is a necessary requisite for the activation of the mechanisms leading to cell cycle arrest and/or apoptotic cell death in cancer cells treated with drugs inhibiting ribosome biogenesis.

There is evidence that this could be mostly true in the case of a normally functioning pRb pathway. Indeed, the absence of pRb could be a major factor conditioning the sensitivity of cancer cells to the exposure of RiBi inhibitors, also when the p53 pathway is dysfunctional [77]. Preliminary studies on this topic were conducted on solid tumor models, such as breast cancer. In fact, the contemporary absence of pRb and functional p53 has been shown to be responsible for a marked reduction of the cell population growth after the inhibition of ribosome biogenesis by actinomycin D, 5-fluorouracyl, methotrexate, and doxorubicin, which was even greater than that observed in p53 proficient cells [64, 78]. The cause of this increased sensitivity lies in the complete abrogation of the two cell cycle checkpoints in the absence of RB [19, 99, 100]: in cells lacking RB, the inhibition of ribosome biogenesis does not hinder the cell cycle progression, thus leading the cells to divide without having reached an appropriate ribosome complement. Very rapidly, the reduction of ribosome complement becomes incompatible with cell survival and a progressive increase of apoptotic cell death occurs [64]. These experimental data are consistent with studies investigating the relationship between the p53 and RB status and its implications on the clinical outcome after treatment with drugs inhibiting ribosome biogenesis. In a series of breast cancers treated with an adjuvant chemotherapeutic protocol including cyclophosphamide, methotrexate, and 5-fluorouracil, the presence of a wild-type or mutated p53, considered independently of the RB status, proved to have a null prognostic value. However, by excluding the cases with no pRb expression or inactivated-hyper-phosphorylated pRb, the p53 status resulted the only factor predicting the patient clinical outcome with patients with wt *TP53* having a much better prognosis compared to those with mutated *TP53*. Worth of noting, the lack of pRb expression was the only independent factor predicting a good clinical outcome in patients treated with adjuvant chemotherapy [101, 102]. Moreover, an RB loss gene expression signature was demonstrated to be associated with increased pathological complete response to neoadjuvant chemotherapy in both estrogen-receptor positive and negative breast cancers [102]. Although the role of pRb pathway has not been evaluated as extensively as p53, similar observations were reported in hematological malignancies. In anaplastic large cell lymphoma, absence of pRb expression was observed in 40% of cases and hyperphosphorylation of pRb was detected in a significant fraction of RB positive patients, consistent with RB inactivation. Notably, these alterations correlated with a favorable clinical outcome [103]. In chronic lymphoid leukemia, 13q14 deletion is a frequent genomic alteration, and although the specific pathogenetic role of *RB1* loss in the context of 13q14 deletion is yet to be determined, this cytogenetic abnormality predicts good clinical outcome following therapy with the FCR (fludarabine, cyclophosphamide, rituximab) regimen [104].

Similarly, trisomy 12 (resulting in copy number gain of CDK4 with consequent hyperphosphorylation and inactivation of pRb) is associated with excellent outcomes following chemoimmunotherapy [104]. Of note, the contemporary presence of 13q14 deletion seems to attenuate the adverse outcome related to the presence of *TP53* deletions in CLL [105]. Since the RB1 locus is affected in less than 50% of CLL cases harboring 13q14 deletions [106], it would be interesting to investigate whether specific loss of *RB1* attenuates the poor prognosis related to *TP53* alterations. In conclusion, these data taken together indicate that (1) the presence of wt p53 associated with a normal downstream pRB pathway is an important characteristic which render cancer cells very sensitive to drugs inhibiting ribosome biogenesis and (2) cancer cells with *RB1* loss could be sensitive to ribosome biogenesis inhibitors irrespective of the p53 status.

However, the integrity of the p53/pRb pathway might not be the only factor affecting response to ribosomal biogenesis inhibition, as described below.

### The rate of ribosome biogenesis of the cell

Other than arresting cell cycle progression, stabilized p53 may cause programmed cell death by inducing transcription of pro-apoptotic factors [63, 65, 66]. Induction of apoptosis by inhibitors of ribosome biogenesis depends on the level of p53 stabilization, apoptosis being activated only by high amount of stabilized p53. In turn, the amount of stabilized p53 was shown to be directly related to the ribosome biogenesis rate of the cell. This was demonstrated by using four drugs, which inhibit rRNA synthesis at different steps: actinomycin D, doxorubicin, 5-fluorouracyl, and CX-5461 [63]. In cells characterized by a high rate of rRNA transcription, the inhibition of ribosome biogenesis caused a significantly greater degree of p53 stabilization and consequent greater expression of the pro-apoptotic members of the *Bcl-2* gene family, *PUMA*, and *BAX*, compared to those characterized by a lower baseline rRNA synthesis. Accordingly, apoptotic cell death occurred in cells with a high rRNA synthesis and not in cells with a low ribosome biogenesis rate, the latter showing only cell cycle arrest. The tight relationship between the level of p53 stabilization and the rRNA synthesis rate was due to the fact that, upon ribosome biogenesis inhibition, different amounts of RPs, no longer used for ribosome building, bind to MDM2, thus hindering with higher efficiency the proteasomal degradation of p53 [63]. Interestingly, in cells with low rRNA synthesis (in which the inhibition of ribosome biogenesis stabilized p53 in a level that was not sufficient for apoptosis induction), the combined treatment with hydroxyurea which activates p53 with a different mechanism allowed to increase the total amount of stabilized p53 inducing apoptotic cell death [55].

Since the induction of cell death, and not cell cycle arrest, is the main goal of cancer chemotherapy, these observations might be relevant for establishing more effective and appropriate therapeutic protocols. In fact, this model implies that ribosome biogenesis inhibitors as single agents could be highly effective in p53 wild-type cancers with a high ribosome biogenesis rate, by inducing apoptotic cell death, whereas for treating cancers with a low ribosome biogenesis rate, they should be combined with drugs capable of stabilizing p53 or inducing apoptosis through different mechanisms. This model applies well in the setting of *TP53* wild-type lymphoproliferative neoplasms, where aggressive lymphomas such as DLBCLs, characterized by high ribosomal biogenesis rates [107], can be cured with standard R-CHOP polychemotherapy [84–86], whereas indolent B cell non-Hodgkin lymphomas (such as small lymphocytic lymphoma/chronic lymphoid leukemia, marginal zone lymphoma, and follicular lymphomas), characterized by low ribosomal biogenesis rates [107], are virtually incurable with the same type of polychemotherapy [89].

### Ribosomal protein deletions and mutations

Since the main mechanism involved in p53 stabilization upon ribosome biogenesis inhibition is represented by the binding of RPs to MDM2, mutations of ribosomal proteins may constitute another factor influencing the response of cancer cells to ribosome biogenesis inhibitors. As reported above, RPL5 and RPL11 play a major role in MDM2 inactivation. However, many other RPs, including RPL3, RPL6, RPL23, RPL26, RPL37, RPS7, RPS14, RPS15, RPS19, RPS20, RPS25, RPS26, and RPS27, have been shown to bind to MDM2, thus stabilizing p53 after induction of ribosomal stress (see for a recent and comprehensive review: [52]). There is increasing evidence for the presence of ribosomal protein copy number changes and mutations in many types of cancer. Regarding the RPs of the large ribosome subunit, exome sequencing demonstrated the presence of mutations of *RPL5* in T cell acute lymphoblastic leukemia (T-ALL) [108] and in glioblastoma [109], and loss of the 1p22.1 region encompassing the *RPL5* gene was found in 20% of multiple myeloma cases (MM) [110]. Furthermore, *RPL5* and *RPL10* mutations were recently observed, even though at low frequency, in MM [111]. The frequency of inactivating *RPL5* mutations and deletions was found to be 11% in glioblastoma, 28% in melanoma, and 34% in breast cancer patients [112]. In T-ALL, *RPL10* and *RPL11* mutations have been also described [108, 113] and *RPL22* was found to be deleted in about 10% patients [114]. *RPL22* mutations were observed to occur with high frequency in endometrial [115, 116] and colorectal cancer [117] with microsatellite instability. Regarding the proteins constituting the small ribosome subunit, whole exome sequencing of chronic lymphocytic leukemia showed recurrent mutations of *RPS15* [117, 118] while mutations of *RPS20* are associated with colorectal carcinoma [119]. There are still few data on the effect of ribosomal protein deletion or mutations on the response to chemotherapeutic treatments. Experiments conducted using cancer cell lines demonstrated that silencing the expression of RPL5 and RPL11 strongly reduced the stabilization and activation of p53 caused by selective rRNA transcription inhibitors [120, 121], suggesting that cancers carrying these genetic changes should be resistant to chemotherapy based on inhibitors of ribosome biogenesis.

Up to now, the only clinical evidence of the impact of RP genetic changes on chemotherapy resistance based on a reduced activation of the RP-MDM2-p53 pathway comes from the study by Ljungström et al. [118] on the relationship between *RPS15* mutations and clinical outcome of patients with chronic lymphocytic leukemia. The authors found that patients with *RPS15* mutations, but carrying wild-type *TP53*, treated with standard chemoimmunotherapy (combination of fludarabine, cyclophosphamide, and rituximab), had a shorter 10-year survival compared with patients without mutated *RPS15*, and an overall survival similar to patients characterized by other adverse-prognostic markers. In the same study, the authors, using a human tumor cell line, demonstrated that transiently expressed mutant *RPS15* reduced the expression of p53 due to an increased ubiquitin-mediated p53 degradation in comparison with cells carrying wild-type RPS15. It could be possible that mutated RPS15 is not capable of neutralizing the MDM2-mediated p53 digestion [122], thus reducing the induction of stabilized p53 upon chemotherapy treatment. In line with these data, our group recently found non-recurrent mutations of multiple RP genes in a significant fraction of DLBCL cases (> 10%) and *RPS12* and *RPL22* deletions in up to 20% of cases. Furthermore, our preliminary data indicate that these alterations are mutually exclusive with *TP53* mutations and that RP mutations could be associated with adverse outcome in *TP53* wild-type patients (manuscript submitted).

In conclusion, although preliminary evidence suggests that RP mutations could provide cancer cells with alternative mechanisms to inactivate p53-mediated responses to nucleolar stress, more studies are needed on the occurrence of RP gene deletions and mutations in cancer cells and their influence on p53 stabilization and therapeutic response after treatment with ribosome biogenesis inhibitors.

### Mutated nucleophosmin

Nucleophosmin (NPM1), also called protein B23, numatrin, and NO38, is a non-ribosomal phosphoprotein, primary located in the nucleolus [123, 124]. NPM1 shuttles between the nucleolus and the cytoplasm [125] and exerts a series of different biochemical functions, some of them being independent of ribosome biogenesis (see for review [126–129]). Regarding the relationship between NPM1 and ribosome biogenesis, there is evidence that NPM1 plays a role in rRNA maturation [130] and its chaperone activity may facilitate the process of ribosome assembly [131]. Furthermore, *NPM1* has been shown to be an important mediator, connecting the BCR-ABL network to ribosome biogenesis and, hence, protein synthesis and cell growth in chronic myelogenous leukemia [132]. Lastly, in proliferating cells, the amount of NPM1 is directly related to the rRNA transcription rate [133] and in human cancer cell lines to the nucleolar size and to the rate of cell proliferation [134].

Quantitative and qualitative changes of NPM1 have been reported to occur in many human malignancies (see for review [126]). Heterozygous NPM1 mutations were observed to occur in about 30% of patients with acute myeloid leukemia (AML) and, with very few exceptions, were restricted to exon 12 [135, 136]. Mutant NPM1 is delocalized to the cytoplasm (NPM1c+) while the amount of wild-type NPM1 located in the nucleolus is reduced as a consequence of haploinsufficiency and formation of heterodimers with mutated NPM1 in the cytoplasm [136]. Importantly, NPM1 mutations are mutually exclusive with TP53 mutations [137] and consistent with this observation the presence of NPM1c+ inhibits p53-mediated responses: in fact cytoplasmic NPM1 localization determines sequestration of ARF tumor suppressor in the cytoplasm, therefore limiting the interaction of ARF with MDM2 with consequent increased p53 degradation [138–140]. It is noteworthy that from the clinical point of view acute myeloid leukemia with mutated NPM1 is characterized by a better prognosis due to a higher remission rate after chemotherapy containing anthracyclines and cytarabine [91, 141]. This is probably due to the fact that leukemic cells with mutated NPM1 maintain a functional wild-type p53 [137]. In line with this data, a recent study reported that patients with AML with mutated NPM1, not eligible for intensive chemotherapy or with refractory or relapsed disease, may be successfully treated with actinomycin D, at the same dose as that used for low-risk gestational trophoblastic tumors [142]. The rationale at the basis of this therapeutic strategy is that leukemic cells with mutated NPM1 may have a more vulnerable nucleolus to the stress induced by the inhibition of ribosome biogenesis, resulting in a very strong p53-mediated response. NPM1 is also a frequent target of chromosomal translocations. The NPM1-ALK (anaplastic lymphoma kinase) fusion protein is the hallmark of ALK-positive anaplastic large cell lymphoma (reviewed in [143]). The NPM1-ALK fusion protein activates a series of cellular signaling pathways boosting lymphomagenesis while inhibiting p53 activity with MDM2 and JNK (c-Jun N-terminal kinase) dependent mechanisms [144]. Therefore, ALK-positive ALCL often retain a functional p53-mediated response to nucleolar stress, and accordingly *TP53* mutations are rare in NPM1-ALK-positive ALCL. In line with these findings, NPM1-ALK-positive ALCL are characterized by a better prognosis following conventional CHOP compared to their ALK negative counterparts. Further investigations on the relationship between the functional state of the nucleolus and the response to ribosome biogenesis inhibitors should be conducted with the aim of establishing therapeutic protocols based on selective inhibition of ribosome biogenesis.

## Conclusions

**Fig. 2:**
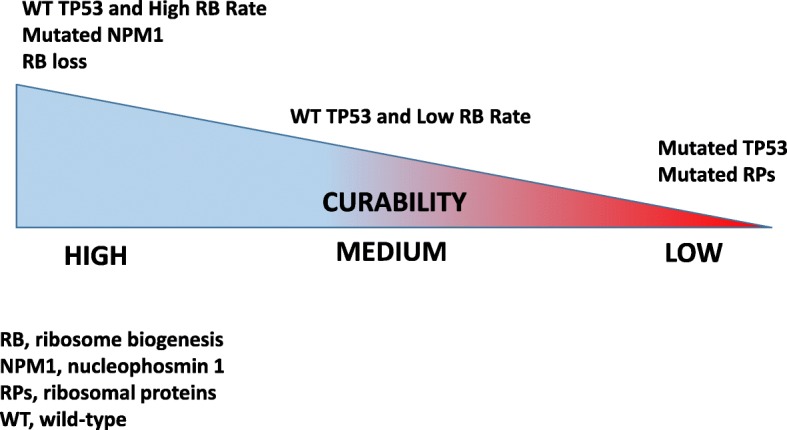
Schematic model representing the relationship between certain intrinsic cancer cell characteristics and curability of hematologic malignancies following chemotherapy based on drugs inhibiting ribosome biogenesis. Cancers with wild-type TP53, high ribosome biogenesis rate, loss of retinoblastoma protein, mutated NPM1 are characterized by good prognosis following chemotherapy (this is the case of TP53 wild-type HL, ALCL, DLBCL, NPM1c+ AML). At the opposite side of the spectrum, cancers characterized by mutant TP53 or mutant ribosomal proteins genes are associated with a low cure rate (certain forms of DLBCL, MM, T-ALL, CLL, AML). In the middle, cancers with low ribosomal biogenesis rate and wild-type TP53 harbor an intermediate cure rate (FL, other indolent B cell lymphoma subtypes)

Despite the advent of personalized medicine, current treatment algorithms do not take into account important biological parameters which have been demonstrated to affect the cancer response to chemotherapeutic agents (these factors are summarized in Table 2) [14–16, 91, 92, 94, 103, 108, 110, 111, 118, 146–161]. There is now evidence that the efficacy of many of the chemotherapeutic drugs used for cancer treatment is related to p53 stabilization consequent to ribosome biogenesis inhibition (Fig. 1), and efforts are ongoing to develop new drugs that can selectively target ribosome biogenesis, without having the genotoxic effects proper of standard chemotherapeutic agents. In this context, it is worth mentioning the selective inhibitor of rRNA transcription, the CX-5461 molecule [7, 75], which may represent a new, very interesting strategy for cancer therapy [12, 162–164]. In this research field, other molecular compounds specifically hindering rDNA transcription have been proposed, demonstrating the increasing interest in this new therapeutic approach [9–11, 165]. On the other hand, as reported in the present review, a series of experimental and clinical data indicate that human tumors are characterized by several genomic alterations determining a highly variable response to the treatment with ribosome biogenesis inhibitors. In fact, several mechanisms converge in attenuating the anticancer activity of ribosome biogenesis inhibitors, mostly by reducing the amount of stabilized p53 and/or the extent of apoptotic responses to RIBi inhibitor-dependent nucleolar stress (Table 2). Accurate knowledge of these mechanisms could provide the rationale for treatment strategies able to by-pass resistance to RIBi inhibitors, such as combinations with MDM2 inhibitors or small molecule inhibitors of phosphatidyl-inositol-3-kinase (PI3K) pathway or antiapoptotic proteins such as bcl-2. The main characteristics influencing the response of hematologic malignancies to drugs inhibiting ribosome biogenesis are summarized in Fig. 2. These characteristics should be considered and evaluated in advance, in order to predict the degree of therapeutic response, especially when using selective inhibitors of ribosome biogenesis.

**Table 2:** Overview of genomic alterations involved in the regulation of the RP/MDM2/p53 axis in hematologic malignancies

Genomic alteration	Disease type	Incidence of the alteration	Prognostic impact	Proposed Mechanism	Reference
TP53 mutation	DLBCL	22%-24%	Poor	Impaired p53 mediated response to nucleolar stress	[14, 146]
	CLL	7-9%	Poor		[94, 147–149]
	ALCL	8%	Poor		[145]
	ALL	14-15%	Poor		[15, 150]
	AML	5%-9%	Poor		[92, 151]
	MM	<5%	Poor		[152]
TP53 deletion	DLBCL	12%	Poor		[16]
	CLL	5-12%	Poor		[147, 148]
	ALL	11%	Poor		[15]
	MM	9.5%	Poor		[152]
ARF deletion	DLBCL	35%	Poor	Increased MDM2-dependent p53 degradation	[153]
	FL	8%	Poor		[154]
	ALL	14-15%	Poor		[15, 150, 155]
RB1 loss	DLBCL	11%	Neutral	Loss of G1/S checkpoint	[156]
	CLL	20%	Neutral		[157]
	ALCL	40%	Good		[103]
	ALL	9%	Neutral		[158, 159]
RPS15 mutation	CLL	19% (RELAPSE)	Poor	Impaired p53 mediated response to nucleolar stress	[118]
RPL5 mutation	MM	Sporadic	NE		[111]
	T-ALL	<5%	NE		[108]
RPL5 deletion	MM	20%	Poor		[110]
RPL10 mutation	T-ALL	5%	NE		[108]
RPL22 deletion	T-ALL	10%	NE		[160]
NPM1 mutation	AML	53%	Good*	Increased sensitivity to nucleolar stress	[91]
NPM1-ALK	ALCL	55%	Good		[161]

Abbreviations: NE (not evaluated), DLBCL (diffuse large B-cell lymphoma), FL (Follicular lymphoma), CLL (chronic lymphoid leukemia), ALCL (anaplastic large T-cell lymphoma), ALL (acute lymphoid leukemia), T-ALL (T-cell acute lymphoid leukemia), MM (Multiple Myeloma), AML (acute myeloid leukemia)

*Associated with good prognosis in the absence of FLT3 genomic alterations

## Abbreviations

ABVD: Doxorubicin, bleomycin, vinblastine, and dacarbazine
ALCL: Anaplastic large cell lymphoma
ALK: Anaplastic lymphoma kinase
ALL: Acute lymphoblastic leukemia
BAX: Bcl-2-associated X protein
Bcl-2: B cell lymphoma 2
CHOP: Cyclophosphamide, doxorubicin, vincristine, and prednisone
DLBCL: Diffuse large B cell lymphoma
DRB: Dichloro-ribofuranosylbenzimidazole
FCR: Fludarabine, cyclophosphamide, rituximab
HL: Hodgkin lymphoma
JNK: c-Jun N-terminal kinase
MDM2: Murine double minute 2
NPM1: Nucleophosmin
Pol I: RNA polymerase I
Pol II: RNA polymerase II
Pol III: RNA polymerase III
pRb: Retinoblastoma protein
PUMA: P53 upregulated modulator of apoptosis
R: Rituximab
RiBi: Ribosome biogenesis
RPLs: Ribosomal proteins of the large subunit
RPs: Ribosomal proteins
RPSs: Ribosomal proteins of the small subunit
SL1: Selectivity factor 1
TIF-I: Transcription initiation factor I
TP53: Tumor protein 53
UBF: Upstream binding factor
